# Acid–base balance, serum electrolytes and need for non-invasive ventilation in patients with hypercapnic acute exacerbation of chronic obstructive pulmonary disease admitted to an internal medicine ward

**DOI:** 10.1186/s40248-016-0063-2

**Published:** 2016-05-25

**Authors:** Alfonso Schiavo, Maurizio Renis, Mario Polverino, Arcangelo Iannuzzi, Francesca Polverino

**Affiliations:** Division of Internal Medicine, Cava de’ Tirreni Hospital, University of Salerno, Cava de’ Tirreni, SA, Italy; Division of Pulmonary Medicine, Scafati Hospital, Salerno, Italy; Division of Internal Medicine, A. Cardarelli Hospital, Naples, Italy; Division of Pulmonary and Critical Care Medicine, Department of Medicine, Brigham and Women’s Hospital, Harvard Medical School, Boston, MA USA

**Keywords:** COPD, Acid–base balance, Hypercapnia, NIV

## Abstract

**Background:**

Hypoventilation produces or worsens respiratory acidosis in patients with hypercapnia due to acute exacerbations of chronic obstructive pulmonary disease (AECOPD). In these patients acid–base and hydroelectrolite balance are closely related. Aim of the present study was to evaluate acid–base and hydroelectrolite alterations in these subjects and the effect of non-invasive ventilation and pharmacological treatment.

**Methods:**

We retrospectively analysed 110 patients consecutively admitted to the Internal Medicine ward of Cava de’ Tirreni Hospital for acute exacerbation of hypercapnic chronic obstructive pulmonary disease. On admission all patients received oxygen with a Venturi mask to maintain arterial oxygen saturation at least >90 %, and received appropriate pharmacological treatment. Non-Invasive Ventilation (NIV) was started when, despite optimal therapy, patients had severe dyspnea, increased work of breathing and respiratory acidosis. Based on Arterial Blood Gas (ABG) data, we divided the 110 patients in 3 groups: A = 51 patients with compensated respiratory acidosis; B = 36 patients with respiratory acidosis + metabolic alkalosis; and C = 23 patients with respiratory acidosis + metabolic acidosis. 55 patients received only conventional therapy and 55 had conventional therapy plus NIV.

**Results:**

The use of NIV support was lower in the patients belonging to group B than in those belonging to group A and C (25 %, vs 47 % and 96 % respectively; *p* < 0.01). A statistically significant association was found between pCO_2_ values and serum chloride concentrations both in the entire cohort and in the three separate groups.

**Conclusions:**

Our study shows that in hypercapnic respiratory acidosis due to AECOPD, differently from previous studies, the metabolic alkalosis is not a negative prognostic factor neither determines greater NIV support need, whereas the metabolic acidosis in addition to respiratory acidosis is an unfavourable element, since it determines an increased need of NIV and invasive mechanical ventilation support.

## Background

Hypercapnic respiratory failure is a complex clinical and functional condition, characterized by an alteration of the acid/base (AB) balance, associated with multi-organ impaired function. Several physiological systems are involved in the control of the AB balance, namely the respiratory system, the kidney, as well as red blood cells and blood proteins, and the bicarbonate buffering system. The AB balance and hydro-electrolytic (HE) balance are closely related, as for any increase in CO_2_ (respiratory acidaemia), a counterbalancing metabolic alkalosis occurs as main compensatory mechanism achieved by a complex ion urinary excretion mechanism [1].

Therefore, in order to manage the AB disorders it is necessary to understand its fine regulatory mechanisms.

For the interpretation of AB disorders two approaches can be used: a physical-chemical approach (which relies on the theory of the Strong Ion Difference [SID]) or a pathophysiologic approach (which relies on the compensation laws). Many authors consider the physicochemical approach a complex and unfeasible approach, and poorly matching with the clinical reality. Moreover, it requires many factors to calculate the SID. On the other hand, the physio-pathological approach is easier and much more reliable, because it provides a quantitative measurement of the AB compensatory responses. Many trials, performed in different clinical disorders, have supported its use in humans [2].

In this study we used the pathophysiologic approach, according to the compensation laws, to evaluate the Chronic Obstructive Pulmonary Disease (COPD) acute exacerbations.

In hypercapnic AECOPD the hypoventilation produces or worsens respiratory acidosis. Since most of these patients, especially if elderly and critical, are multi-drugs recipients for comorbidities, the AB and HE disorders are very common, producing a potential bias in the interpretation of the final values [3].

Thus, we sought to evaluate: 1) the AB and HE disorders in hypercapnic AECOPD patients; and 2) the effect of the type of treatment (pharmacological or by non-invasive ventilation [NIV]) on the AB and HE disorders,

## Methods

We retrospectively analysed the data of inpatients admitted in 2012 to the Internal Medicine Division of Cava de’ Tirreni Hospital (Italy) for hypercapnic AECOPD.

COPD and AECOPD were defined according to the GOLD guidelines [4]. Hypercapnia was defined as PaCO_2_ > 45 mmHg [4].

Between January and December 2012, 140 patients were admitted for hypercapnic AECOPD. Thirty patients were excluded for the following reasons: 10 patients were on NIV home care, 7 had a pneumonia, 3 had ARDS, 10 had severe respiratory failure (pH < 7.1, hypercapnic coma or hemodynamic instability) and were admitted to the Intensive Care Unit (ICU). Information about comorbidities were obtained from the primary care physicians.

Anthropometric characteristics, clinical parameters, haematological and biochemical exams, and blood gas analyses were assessed upon admission. The Glasgow Coma Scale was used for the state of consciousness [5].

Patients were classified into three categories: A = compensated respiratory acidosis; B = respiratory acidosis with metabolic alkalosis; and C = respiratory acidosis with metabolic acidosis [6].

On admission all patients received oxygen with a Venturi mask to maintain arterial oxygen saturation (SaO_2_) at least >90 %, and received treatment, besides the treatments for the comorbidities, with conventional therapy: nebulized bronchodilators, systemic glucocorticoids, and antibiotics [7].

NIV was started when the oxygen therapy and optimal pharmacological treatment did not produce any clinical improvement and patients had severe dyspnea, increased work of breathing and respiratory acidosis (pH <7.35 or arterial carbon dioxide pressure > 45 mmHg or both). All patients were conscious and able to protect their airway and deal with respiratory secretions.

NIV was performed through oronasal mask with a pressure/volume ventilator (Carat II, Hoffrichter). Support pressure, Positive End Expiratory Pressure (PEEP) and triggered flows were adjusted to obtain a tidal volume of 6–8 ml/kg, the best possible oxygenation, and a decrease in respiratory rate. Support pressure and PEEP were modified based on the arterial gases. The mean Inspiratory Positive Assisted Pressure (IPAP) was 16 ± 4 cmH_2_O. None of the patients refused the treatment or required discontinuation of ventilation for discomfort, NIV was suspended when clinical steady-state was achieved: respiratory rate <24/min, heart rate <110/beats-min, pH >7.35 and SaO_2_ > 90 % with FiO_2_ < 40 % [8].

Patients were transferred to ICU for invasive mechanical ventilation if the following conditions occurred: 1) worsening of arterial pH and PaCO_2_; 2) clinical signs of pre-coma; 3) cardiovascular instability [9].

Based on ABG data, according to the compensation laws, we divided the 110 patients in the 3 groups previously identified: A = 51 patients. with compensated respiratory acidosis; B = 36 patients with respiratory acidosis + metabolic alkalosis; and C = 23 patients. with respiratory acidosis + metabolic acidosis.

### Statistical analysis

All quantitative variables are reported as mean ± standard deviation (SD).

Statistical analyses were conducted using parametric or nonparametric tests, based on the continuous or asymmetrical distribution of the variables. Student’s *T* test for unpaired data was used to compare the quantitative variables between the two groups: patients in NIV support (NIV+) vs patients treated with conventional therapy (NIV-). The *χ*2 test was used to compare ordinal variables.

The odds ratio (OR) for NIV+ vs NIV- and the 95 % confidence interval (95 % CI) were calculated using a logistic regression analysis for the 3 categories of ABG disorders, and group B (respiratory acidosis + metabolic alkalosis), which showed the lower percentage of NIV support, was used as reference group.

Linear regression analysis was used to evaluate the association between pCO_2_ and blood electrolytes.

The statistical analysis was performed using Statistical Package for Social Science (SPSS 12).

## Results

The patients with COPD and hypercapnia had the following comorbidites: chronic ischaemic heart disease (35 %); atrial fibrillation (15 %); heart failure (32 %); previous stroke (11 %); diabetes mellitus (26 %); chronic renal failure (44 %). Twenty-eight percent of patients had peripheral edema.

The vast majority of patients was receiving cardiovascular therapy: diuretics (86 %), potassium-sparing diuretics (6 %), ACE inhibitors (25 %), angiotensin receptor antagonists (29 %), digoxin (9 %), selective beta blockers (6 %), calcium channel blockers (21 %), nitro-derivatives (10 %), as well as antidiabetic medications, aspirin, statins and inhaled bronchodilators.

Fifty-five subjects required NIV. In 33 patients out of the 55, the acidosis was corrected by the NIV, whereas the remaining 22, in which the acidosis persisted, were transferred to ICU. The mean NIV time, in successfully treated patients, was 7.8 ± 5.0 days.

### NIV+ vs NIV-

Anthropometric data, clinical, biochemical and ABG parameters are shown in Table 1. Patients were divided into two groups: standard therapy (NIV -) and standard therapy plus NIV (NIV +).

**Table 1:** Clinical and metabolic parameters (mean ± SD)

*Variables*	*NIV- (N = 55)*	*NIV+ (N = 55)*	*p*
Age (years)	77.5 ± 8.8	77.6 ± 10.7	0.94
Gender (M/F)	29/26	28/27	0.85
Glucose (mg/dl)	142.3 ± 48.9	156.2 ± 76.5	0.26
Serum Uric Acid (mg/dl)	6.3 ± 2.0	6.6 ± 2.6	0.60
Serum Albumin (g/dl)	3.3 ± 0.5	2.9 ± 0.5	<0.001
C-Reactive-Protein (mg/l)	76.9 ± 75.7	27.4 ± 34.4	0.039
Serum Creatinine (mg/dl)	1.08 ± 0.58	1.29 ± 0.88	0.14
GFR (MDRD ml/min/1.73 m^2^)	71.8 ± 32.0	72.7 ± 49.3	0.91
HR (bpm)	83.8 ± 12.7	93.6 ± 13.6	<0.001
SBP (mmHg)	124.1 ± 17.0	120.0 ± 17.9	0.21
DBP (mmHg)	74.2 ± 9.9	72.5 ± 10.2	0.38
PaO_2_ (mmHg)	58.4 ± 13.5	60.2 ± 16.2	0.54
PaO_2_/FiO2	255.9 ± 38.6	200.2 ± 57.6	<0.001
pH	7.42 ± 0.05	7.29 ± 0.11	<0.001
PaCO_2_ (mmHg)	50.5 ± 5.5	75.5 ± 19.8	<0.001
Lactate (mmol/L)	1.38 ± 0.68	2.01 ± 1.76	0.017
Serum HCO3^−^ (mmol/L)	32.6 ± 5.5	36.0 ± 10.1	0.036
Serum Na^+^ (mmol/L)	138.6 ± 3.9	139.1 ± 6.9	0.63
Serum K^+^ (mmol/L)	4.4 ± 0.6	4.7 ± 0.8	0.037
Serum Ca^++^ (mmol/L)	8.6 ± 0.6	8.4 ± 0.8	0.12
Serum Mg^+^ (mmol/L)	1.96 ± 0.32	1.89 ± 0.32	0.24
Serum Cl^−^ (mmol/L)	99.4 ± 3.9	96.6 ± 8.1	0.030

*GFR* Glomerular Filtration Rate, *MDRD* Modification of Diet in Renal Disease

Compared to NIV -, patients of NIV + had, in addition to higher PaCO_2_ and lower values of pH and PaO_2_/FiO_2_, increased heart rate, higher values of blood potassium, bicarbonates and lactates and lower albumin and serum chloride concentrations (Table 1).

### NIV and ABG groups

The use of NIV support was lower in the patients belonging to group B (respiratory acidosis + metabolic alkalosis) than those belonging to group A (compensated respiratory acidosis) and group C (respiratory acidosis + metabolic acidosis, 25 %, vs 47 % and 96 % respectively; *p* < 0.01) (Table 2).

**Table 2:** Non-Invasive-Ventilation (NIV +) in groups of patients according to ABG analysis values

	Mixed respiratory acidosis – metabolic alkalosis (*N* = 36)	Compensated respiratory acidosis (*N* = 51)	Mixed respiratory - metabolic acidosis (*N* = 23)
NIV -	75 % (27)	53 % (27)	4 % (1)
NIV +	25 % (9)	47 % (24)	96 % (22)

*χ* ^2^ < 0.001

Using a logistic regression model, we observed that the probability of requiring NIV, in comparison to the reference group B, was approximately 3 times higher in patients of group A (OR 2.7; 95 % CI 1.04–6.82; p <0.05) and 67 times higher in patients of group C (OR 67.1; 95 % CI 7.9–572.8; *p* < 0.001).

### Electrolytes, edema and medications in the 3 groups

In a variance analysis, a statistically significant difference in serum albumin concentrations was found in group C (2.7 ± 0.11 g/dL) compared to group B (3.2 ± 0.09 g/dL; *p* = 0.012) and group A (3.2 ± 0.07 g/dL; *p* = 0.003).

Significant hyperlactatemia (lactate values ≥ 2 mmol/L) was found in all groups, even though in different percentages: in group B, patients with symptomatic hyperlactatemia were all in conventional therapy (6/20: 33 % of subjects in NIV- and no one in NIV +); in the group A: 4/20 (25 %) in NIV - and 6/18 (33.3 %) in NIV +, and in the group C: 11/22 (50 %) in NIV +, and no one in NIV -.

The incidence of peripheral edema was not statistically different between the 3 ABG groups: 25 % in group B, 26 % in group A, and 47 % in group C.

A statistically significant association was found between pCO_2_ values and serum chloride concentrations both in the entire cohort and in the three separate groups (Table 3).

**Table 3:** Linear association among pCO_2_ and serum electrolytes in groups of patients according to ABG analysis values and in the entire cohort

Variables		Mixed respiratory acidosis – metabolic alkalosis (*N* = 36)	Compensated respiratory acidosis (*N* = 51)	Mixed respiratory - metabolic acidosis (*N* = 23)	Hypercapnic COPD, entire group (*N* = 110)
Na^+^	R^2^	0.10	0.01	0.16	0.002
	Slope	0.67	1.06	−1.33	−0.02
	p	0.06	0.03	0.06	0,62
K^+^	R^2^	0.001	−0.02	−0.001	0.003
	Slope	−1.53	−0.66	−8.08	1.45
	p	0.58	0,84	0.33	0.58
HCO_3_^−^	R^2^	0.84	0.90	0.49	0.24
	Slope	1.26	2.18	2.20	1.14
	p	0.001	0.001	0.001	0.001
Cl^−^	R^2^	0.30	0.32	0.21	0.20
	Slope	−1.28	−1.45	−1.24	−1.37
	p	0.001	0.001	0.04	0.001
Anion Gap	R^2^	0.20	0.11	0.08	0.04
	Slope	−0.71	−1.39	−0.82	−0.67
	p	0.006	0.01	0.23	0.03
Lactate	R^2^	0.10	0.02	0.01	0.01
	Slope	−5.54	−3.42	−0.95	1.58
	p	0.06	0.30	0.65	0.25

Table 4 shows the list of medication taken for cardiovascular conditions.

**Table 4:** Cardiovascular drugs in groups of patients according to ABG analysis values and in the entire cohort

	Mixed respiratory acidosis – metabolic alkalosis (*N* = 36)	Compensated respiratory acidosis (*N* = 51)	Mixed respiratory - metabolic acidosis (*N* = 23)	Hypercapnic COPD, entire cohort (*N* = 110)
Diuretics	33/36	40/51	22/23	95/110
ACE inhibitors	9/36	14/50	3/20	26/106
Angiotensin receptor antagonists	12/36	15/50	4/20	31/106
Calcium channel blocking agents	5/36	15/50	3/20	23/106
Beta-blockers	3/36	4/50	0/20	7/106
Nitroglycerin	7/36	3/50	1/20	11/106
Potassium sparing agents	4/36	3/50	1/21	8/107

## Discussion

The hypercapnic AECOPD is an alarming event that requires a careful management. Several factors determine the final outcome in terms of both survival and need for assisted mechanical ventilation in ICU. These factors are patient’s age, general health status, disease severity, hemodynamic stability, concomitant comorbidities, pulmonary function, respiratory acidosis degree, and underlying AB and HE balance disorders [10].

This is a retrospective study, in which patients with hypercapnic AECOPD, managed in an internal medicine ward of a General Hospital have been studied. We have shown that the group of patients with respiratory acidosis + metabolic acidosis (group C) had the most serious prognosis, both in terms of non-invasive ventilation, and ICU support. These patients often underestimate their clinical status so that an acute event easily leads to metabolic derangement, most of the times caused by renal failure or worsening of diabetes mellitus. However, in some patients, adequate medical treatment of the metabolic imbalance, and the correction of hypoxemia and hypercapnia by oxygen and NIV could allow a safe management with quick recovery. In patients with hypercapnic AECOPD, a good metabolic compensation and an adequate renal function significantly reduce the mortality [11].

In respiratory acidosis, if an excessive increase of bicarbonate concentration (higher than expected by renal compensation laws) occurs, a mixed disorder with autonomous metabolic alkalosis should be suspected. In patients with multiple comorbidities and multi-drug treatment, several circumstances could lead to metabolic alkalosis. In our patients, the use of diuretics and steroids and a relative reduction in circulating blood volume were the main causes of metabolic alkalosis. We observed that patient belonging to group B (respiratory acidosis + metabolic alkalosis) had a better prognosis than patients in group A (compensated respiratory acidosis) with a significantly lower need of NIV.

The admission to ICU was not significantly different in comparison to patients belonging to group A.

These data are conflicting with Terzano et al. who observed that metabolic alkalosis worsened the prognosis of respiratory acidosis in hypercapnic AECOPD patients. In their study, patients with mixed disorder (respiratory acidosis plus metabolic alkalosis) had a longer and higher incidence of NIV support than patients with compensated respiratory acidosis [12].

To explain such different finding, we can speculate that patients with hypercapnic AECOPD managed in an Internal Medicine ward of a General Hospital are quite different from patients managed in a Pulmonary Department of a Teaching Hospital. The latter have scheduled follow up visits and are hospitalized only when the clinical status progressively deteriorates. In contrast, hypercapnic AECOPD patients admitted to an Internal Medicine ward of a General Hospital usually come from the Emergency Department, where they are hospitalized because their clinical status worsened for sudden events (such as diarrhoea, vomiting, fever, sweating, decreased fluid intake) with additional autonomous metabolic alkalosis. Therefore, a diuretic and/or glucocorticoid induced metabolic alkalosis with hypovolemia is often detectable in these patients. These events can be easily managed by blood volume and serum chloride restoring (with saline solution) allowing the clinical improvement and avoiding the NIV support.

In an earlier study, it was shown that in COPD patients with peripheral edema treated with loop diuretics, furosemide withdrawing led to increased ventilation and an improvement in the respiratory acidosis [13].

Lactate concentration was higher in NIV+ group. Our result confirms previous studies in which blood lactate was considered a diagnostic hallmark of tissue hypoxia and respiratory muscle fatigue and, indirectly, of COPD severity in patients not requiring NIV [12]. However, hyperlactataemia (lactate values > 2 mmol/L), plays a different role in the three groups of AB disorders. In fact, in group B it was present only in patients not requiring NIV (33 %); in group A was present both in patients requiring (25 %) and not requiring NIV (30 %); and in the group C it was present in 50 % of patients requiring NIV. In group B the lactate overproduction may reflect a positive response to the stress enabling an alternative energetic way through a carbon units transport between cells (“good lactate”); whereas in group C the lactate hyperproduction is probably due to hypoperfusion (“bad lactate”) and should be immediately recognized and treated [14, 15]. In the patients of the NIV-treated respiratory acidosis group we didn’t observe metabolic “post-hypercapnic alcalosis” since the concomitant metabolic component was simultaneously treated and corrected.

We acknowledge that many of the cross-associations between pCO_2_ and serum electrolytes found in our study are expected, as the AB balance is closely related to the HE balance. Nevertheless, some remarks seem worthy of focus.

In Brackett’s classic experiment, on healthy volunteers, acute hypercapnia was experimentally induced by having them hyperventilating by breathing CO_2_–enriched (at 7 % and at 10 %) air. In these subjects, a variation of both pH and bicarbonates occurred, with a very slight, but significant, increase in Na^+^ and K^+^, and no changes in Cl^−^, indeterminate anions (anion gap: AG) and lactates concentrations [16]. In a following study with patients with alveolar hypoventilation and absence of clinical conditions potentially responsible for metabolic alkalosis, the response to the chronic hypercapnia was investigated. In this group a decrease in pH with a progressive increase in pCO_2_ was seen; moreover, when pCO_2_ values increased, Cl^−^ decreased whereas no significant association was found with HE balance (Na^+^, K^+^ and AG) [17].

In our study population composed by patients with hypercapnic AECOPD, we found a significant association between increase in pCO_2_ and bicarbonate (which reflects the renal compensation) and a statistically significant relationship between the pCO_2_ increase and serum chloride decrease. Such associations were present in all the subjects studied and was maintained also when dividing the subjects in the 3 subgroups classified according to the ABG disorder. Serum chloride decrease may be related to bicarbonate increase as an attempt to assure the electro-neutrality. We can interpret the data of serum chloride reduction also in the light of the Strong Ion Difference (SID) of Stewart’s theory (Stewart). In this case, in hypercapnic AECOPD, after the pulmonary response to increasing pCO_2,_ the renal response starts, by regulating Na^+^ and Cl^−^, which are the major SID determinants. A relative (compared to Na^+^ concentration) hypochloremia would lead to a SID increase and would indicate a metabolic alkalosis with a bicarbonate increase. According to this theory, therefore, bicarbonate increase reflects a shift in bicarbonate equilibrium of the ratio H^+/ ^HCO3^−^connected with the SID variation [18]. In fact, in the overall group and in the subgroups with compensated respiratory acidosis and respiratory acidosis + metabolic alkalosis, an inverse association between pCO_2_ and indeterminate anions was found.

## Conclusions

In conclusion our study, performed in a population of inpatients admitted to an Internal Medicine Division for hypercapnic AECOPD, shows that:

1. The AB balance and the HE balance, in agreement with previous studies, are closely interconnected;
2. The electrolytic changes are usually in a range that could be easily corrected with the conventional pharmacological therapy;
3. In hypercapnic respiratory acidosis due to AECOPD, differently from previous studies, the metabolic alkalosis is not a negative prognostic factor neither determines greater NIV support need;
4. The metabolic acidosis in addition to respiratory acidosis is an unfavourable element, since it determines an increased need of NIV and invasive mechanical ventilation support.
